# Supplementary material for: Cost-Effectiveness of the Pneumococcal Vaccine in the Adult Population: A Systematic Review
Source: Healthcare (Basel). 2024 Dec 9;12(23):2490. doi: 10.3390/healthcare12232490 (PMC11641157; doi:10.3390/healthcare12232490)
Supplement: Supplementary file 1 [file healthcare-12-02490-s001.zip › healthcare-3325989-supplementary.pdf]

Table S1. Quality assessment of selected articles according to CHEERs 2022 criteria.

N	Item	Igarashi et al. [1]	Gouveia et al. [2]	Ngamprasertchai et al. [3]	Sun et al. [4]	Wolff et al. [5]	Malene B et al. [6]	Choi et al. [7]	Olsen et al. [8]	Wateska et al. [9]	Mendes et al. [10]	Nymark et al. [11]	Marbaix et al. [12]
1	Title	0.5	1	1	1	0.5	1	1	1	0.5	1	0.5	1
2	Abstract	1	1	1	1	1	1	1	1	1	1	1	1
3	Background and objective	1	1	1	1	1	1	1	1	1	1	1	1
4	Health economic analysis plan	1	1	1	1	1	1	1	1	1	1	1	1
5	Study population	1	1	1	1	1	1	1	1	1	1	1	1
6	Setting and location	1	1	1	1	1	1	1	1	1	1	1	1
7	Comparators	1	1	1	1	1	1	1	1	1	1	1	1
8	Perspective	1	1	1	0.5	1	1	1	0.5	0.5	1	1	1
9	Time horizon	1	0.5	1	0.5	1	1	0.5	1	0.5	0.5	1	0.5
10	Discount rate	1	1	0.5	1	0.5	1	0.5	1	0.5	0.5	1	1
11	Selection of outcomes	1	1	1	1	1	1	1	1	1	1	1	1
12	Measurement of outcomes	1	1	1	1	1	1	1	1	1	1	1	1
13	Valuation of outcomes	1	1	1	1	1	1	1	1	1	1	1	1
14	Measurement and valuation of resources and costs	1	1	1	1	1	1	1	1	1	1	1	1
15	Currency, price date, and conversion	1	1	1	1	1	1	1	1	1	1	1	1
16	Rationale and description of model	1	1	1	1	1	1	1	1	1	1	1	1
17	Analytics and assumptions	1	0.5	1	1	0.5	0.5	1	0.5	1	1	1	1
18	Characterizing heterogeneity	0	0	0	0	0	0	0	0	0	0	0	0
19	Characterizing distributional effect	0	0	0	0	0	0	0	0	0	0	0	0
20	Characterizing uncertainty	1	1	1	1	1	1	1	1	1	1	1	1
21	Approach to engagement with patients and others affected by the study	0	0	0	0	0	0	0	0	0	0	0	0
22	Study parameters	1	1	1	1	1	1	1	1	1	1	1	1

23	Summary of main results	1	1	1	1	1	1	1	1	1	1	1	1
24	Effect of uncertainty	1	1	1	1	1	1	1	1	1	1	1	1
25	Effect of engagement with patients and others affected by the study	0	0	0	0	0	0	0	0	0	0	0	0
26	Study findings, limitations, generalizability, and current knowledge	1	1	1	1	1	1	1	1	1	1	1	1
27	Source of funding	1	1	1	1	1	1	1	1	1	1	1	1
28	Conflicts of interest	1	1	1	1	1	1	1	1	1	1	1	1
	**Total score**	**23.5**	**23**	**23.5**	**23**	**22.5**	**23.5**	**23**	**23**	**22**	**23**	**23.5**	**23.5**
	**Quality ranking**	**High**	**High**	**High**	**High**	**High**	**High**	**High**	**High**	**High**	**High**	**High**	**High**

N	Item	Cantarero et al. [13]	Polistena et al. [14]	Smith et al. [15]	Smith et al. [16]	Thorrington et al. [17]	Wateska et al. [18]	Boer et al. [19]	Restivo et al. [20]	Gourzoulidis et al. [21]	Atwood et al. [22]	Giglio et al. [23]
1	Title	1	1	0.5	0.5	0.5	0.5	1	0.5	1	1	0.5
2	Abstract	1	1	1	1	1	1	1	1	1	1	1
3	Background and objective	1	1	1	1	1	1	1	1	1	1	1
4	Health economic analysis plan	1	1	1	1	1	1	1	1	1	1	1
5	Study population	1	1	1	1	1	1	1	1	1	1	1
6	Setting and location	1	1	1	1	1	1	1	1	1	1	1
7	Comparators	1	1	1	1	1	1	1	1	1	1	1
8	Perspective	1	0.5	1	0.5	1	0.5	1	0.5	1	0.5	0
9	Time horizon	0.5	0.5	0.5	0.5	1	0.5	0.5	1	1	1	0.5
10	Discount rate	1	0.5	0.5	0.5	1	0.5	1	1	1	1	0.5
11	Selection of outcomes	1	1	1	1	1	1	1	1	1	1	0
12	Measurement of outcomes	1	1	1	1	1	1	1	1	1	1	0
13	Valuation of outcomes	1	1	1	1	1	1	1	1	1	1	0
14	Measurement and valuation of resources and costs	1	1	1	1	1	1	1	1	1	1	0
15	Currency, price date, and conversion	1	1	1	1	1	1	1	1	1	1	0.5
16	Rationale and description of model	1	1	1	1	1	1	1	1	1	1	0.5

17	Analytics and assumptions	0.5	1	1	1	1	1	1	1	1	0.5	0
18	Characterizing heterogeneity	0	0	0	0	0	0	0	0	0	0	0
19	Characterizing distributional effect	0	0	0	0	0	0	0	0	0	0	0
20	Characterizing uncertainty	1	1	1	1	1	1	1	1	1	1	1
21	Approach to engagement with patients and others affected by the study	0	0	0	0	0	0	0	0	0	0	0
22	Study parameters	1	1	1	1	1	1	1	1	1	1	1
23	Summary of main results	1	1	1	1			1	1	1	1	1
24	Effect of uncertainty	1	1	1	1	1	1	1	1	1	1	1
25	Effect of engagement with patients and others affected by the study	0	0	0	0	0	0	0	0	0	0	0
26	Study findings, limitations, generalizability, and current knowledge	1	1	1	1	1	1	1	1	1	1	1
27	Source of funding	1	1	1	1	1	1	1	1	1	1	1
28	Conflicts of interest	1	1	1	1	1	1	1	1	1	1	1
	**Total score**	**23**	**22.5**	**22.5**	**22**	**23.5**	**22**	**23.5**	**23**	**24**	**23**	**15.5**
	**Quality ranking**	**High**	**High**	**High**	**High**	**High**	**High**	**High**	**High**	**High**	**High**	**Moderate**

References

1. Igarashi A, Hirose E, Kobayashi Y, Yonemoto N, Lee B. Cost-effectiveness analysis for PCV13 in adults 60 years and over with underlying medical conditions which put them at an elevated risk of pneumococcal disease in Japan. *Expert Rev Vaccines*. Sep 2021;20(9):1153-1165. doi:10.1080/14760584.2021.1952869
2. Gouveia M, Jesus G, Inês M, Costa J, Borges M. Cost-effectiveness of the 13-valent pneumococcal conjugate vaccine in adults in Portugal versus "no vaccination" and versus vaccination with the 23-valent pneumococcal polysaccharide vaccine. *Hum Vaccin Immunother*. 2019;15(4):850-858. doi:10.1080/21645515.2018.1560769
3. Ngamprasertchai T, Kositamongkol C, Lawpoolsri S, et al. A cost-effectiveness analysis of the 13-valent pneumococcal conjugated vaccine and the 23-valent pneumococcal polysaccharide vaccine among Thai older adult. *Front Public Health*. 2023;11:1071117. doi:10.3389/fpubh.2023.1071117
4. Sun X, Tang Y, Ma X, et al. Cost-Effectiveness Analysis of 23-Valent Pneumococcal Polysaccharide Vaccine Program for the Elderly Aged 60 Years or Older in Shanghai, China. *Front Public Health*. 2021;9:647725. doi:10.3389/fpubh.2021.647725
5. Wolff E, Storsaeter J, Örtqvist Å, et al. Cost-effectiveness of pneumococcal vaccination for elderly in Sweden. *Vaccine*. Jul 6 2020;38(32):4988-4995. doi:10.1016/j.vaccine.2020.05.072
6. Malene BM, Oyvind H, Tor M, et al. Cost-effectiveness of 20-valent pneumococcal conjugate vaccine compared with 23-valent pneumococcal polysaccharide vaccine among adults in a Norwegian setting. *Cost Eff Resour Alloc*. Aug 9 2023;21(1):52. doi:10.1186/s12962-023-00458-4
7. Choi MJ, Kang SO, Oh JJ, Park SB, Kim MJ, Cheong HJ. Cost-effectiveness analysis of 13-valent pneumococcal conjugate vaccine versus 23-valent pneumococcal polysaccharide vaccine in an adult population in South Korea. *Hum Vaccin Immunother*. 2018;14(8):1914-1922. doi:10.1080/21645515.2018.1456602
8. Olsen J, Schnack H, Skovdal M, Vietri J, Mikkelsen MB, Poulsen PB. Cost-effectiveness of 20-valent pneumococcal conjugate vaccine in Denmark compared with PPV23. *J Med Econ*. Jan-Dec 2022;25(1):1240-1254. doi:10.1080/13696998.2022.2152235
9. Wateska AR, Nowalk MP, Lin CJ, et al. Cost-Effectiveness of Pneumococcal Vaccination Policies and Uptake Programs in US Older Populations. *J Am Geriatr Soc*. Jun 2020;68(6):1271-1278. doi:10.1111/jgs.16373
10. Mendes D, Averin A, Atwood M, et al. Cost-effectiveness of using a 20-valent pneumococcal conjugate vaccine to directly protect adults in England at elevated risk of pneumococcal disease. *Expert Rev Pharmacoecon Outcomes Res*. Dec 2022;22(8):1285-1295. doi:10.1080/14737167.2022.2134120
11. Nymark LS, Dag Berild J, Lyngstad TM, et al. Cost-utility analysis of the universal pneumococcal vaccination programme for older adults in Norway. *Hum Vaccin Immunother*. Nov 30 2022;18(6):2101333. doi:10.1080/21645515.2022.2101333
12. Marbaix S, Mignon A, Taelman A, Averin A, Atwood M, Vietri J. Cost-utility of 20-valent pneumococcal conjugate vaccine compared to no vaccination and recommended alternative vaccines among Belgian adults. *Expert Rev Vaccines*. Jan-Dec 2023;22(1):1008-1021. doi:10.1080/14760584.2023.2273892
13. Cantarero D, Ocaña D, Onieva-García M, et al. Cost-utility analysis of the use of the 20-valent anti-pneumococcal vaccine (PCV20) in adults older than 60 years in Spain. *Vaccine*. Aug 14 2023;41(36):5342-5349. doi:10.1016/j.vaccine.2023.07.016
14. Polistena B, Icardi G, Orsi A, Spandonaro F, Di Virgilio R, d'Angela D. Cost-Effectiveness of Vaccination with the 20-Valent Pneumococcal Conjugate Vaccine in the Italian Adult Population. *Vaccines (Basel)*. Nov 28 2022;10(12)doi:10.3390/vaccines10122032
15. Smith KJ, Wateska AR, Nowalk MP, et al. Higher-Valency Pneumococcal Conjugate Vaccines: An Exploratory Cost-Effectiveness Analysis in U.S. Seniors. *Am J Prev Med*. Jul 2021;61(1):28-36. doi:10.1016/j.amepre.2021.01.023
16. Smith KJ, Wateska AR, Nowalk MP, et al. Cost-Effectiveness of Newly Recommended Pneumococcal Vaccination Strategies in Older Underserved Minority Adults in the USA. *Infect Dis Ther*. Aug 2022;11(4):1683-1693. doi:10.1007/s40121-022-00669-x
17. Thorrington D, van Rossum L, Knol M, et al. Impact and cost-effectiveness of different vaccination strategies to reduce the burden of pneumococcal disease among elderly in the Netherlands. *PLoS One*. 2018;13(2):e0192640. doi:10.1371/journal.pone.0192640
18. Wateska AR, Nowalk MP, Lin CJ, et al. An intervention to improve pneumococcal vaccination uptake in high risk 50-64 year olds vs. expanded age-based recommendations: an exploratory cost-effectiveness analysis. *Hum Vaccin Immunother*. 2019;15(4):863-872. doi:10.1080/21645515.2018.1564439
19. de Boer PT, van Werkhoven CH, van Hoek AJ, et al. Higher-valency pneumococcal conjugate vaccines in older adults, taking into account indirect effects from childhood vaccination: a cost-effectiveness study for the Netherlands. *BMC Med*. Feb 16 2024;22(1):69. doi:10.1186/s12916-024-03277-3
20. Restivo V, Baldo V, Sticchi L, et al. Cost-Effectiveness of Pneumococcal Vaccination in Adults in Italy: Comparing New Alternatives and Exploring the Role of GMT Ratios in Informing Vaccine Effectiveness. *Vaccines (Basel)*. Jul 18 2023;11(7)doi:10.3390/vaccines11071253
21. Gourzoulidis G, Barmpouni M, Kossyvaki V, Vietri J, Tzanetakos C. Health and economic outcomes of 20-valent pneumococcal conjugate vaccine compared to 15-valent pneumococcal conjugate vaccine strategies for adults in Greece. *Front Public Health*. 2023;11:1229524. doi:10.3389/fpubh.2023.1229524

22. Atwood M, Beausoleil L, Breton MC, Laferriere C, Sato R, Weycker D. Cost-effectiveness of alternative strategies for use of 13-valent pneumococcal conjugate vaccine (PCV13) in Canadian adults. *Can J Public Health*. Dec 2018;109(5-6):756-768. doi:10.17269/s41997-018-0050-9
23. Giglio ND, Castellano VE, Mizrahi P, Micone PV. Cost-Effectiveness of Pneumococcal Vaccines for Adults Aged 65 Years and Older in Argentina. *Value Health Reg Issues*. Mar 2022;28:76-81. doi:10.1016/j.vhri.2021.08.003
